# Prescribing Patterns and Outcomes of Edoxaban in Atrial Fibrillation: One-Year Data from the Global ETNA-AF Program

**DOI:** 10.3390/jcm12051870

**Published:** 2023-02-27

**Authors:** Tze-Fan Chao, Martin Unverdorben, Paulus Kirchhof, Yukihiro Koretsune, Takeshi Yamashita, Robert A. Crozier, Ladislav Pecen, Cathy Chen, Amanda P. Borrow, Raffaele De Caterina

**Affiliations:** 1 Division of Cardiology, Department of Medicine, Taipei Veterans General Hospital, Taipei 11220, Taiwan; 2 Institute of Clinical Medicine, Cardiovascular Research Center, National Yang Ming Chiao Tung University, Taipei 11217, Taiwan; 3 Global Specialty Medical Affairs, Daiichi Sankyo, Inc., Basking Ridge, NJ 07920, USA; 4 Department of Cardiology, University Heart and Vascular Centre Hamburg, University Medical Center Hamburg-Eppendorf, 20246 Hamburg, Germany; 5 German Center for Cardiovascular Research (DZHK), Partner Site Hamburg/Kiel/Lübeck, 20246 Hamburg, Germany; 6 Institute of Cardiovascular Sciences, University of Birmingham, IBR 136, Wolfson Drive, Birmingham B15 2TT, UK; 7 Cardiovascular Division, National Hospital Organization Osaka National Hospital, Osaka 540-0006, Japan; 8 Department of Cardiovascular Medicine, The Cardiovascular Institute, Tokyo 106-0031, Japan; 9 Institute of Computer Science, Academy of Sciences of the Czech Republic, 18207 Prague, Czech Republic; 10 Pisa University Hospital, University of Pisa, 56126 Pisa, Italy; 11 Fondazione VillaSerena per la Ricerca, 65013 Città Sant’Angelo, Italy

**Keywords:** edoxaban, non-vitamin K antagonist oral anticoagulants, dosing, ETNA-AF, registry, real-world data

## Abstract

Non-recommended dosing occurs in ~25–50% of non-vitamin K antagonist oral anticoagulant prescriptions, with limited data for edoxaban. We analyzed edoxaban dosing patterns in atrial fibrillation patients from the Global ETNA-AF program, relating patterns to baseline characteristics and 1-year clinical outcomes. The following dosing groups were compared: non-recommended 60 mg (“overdosed”) vs. recommended 30 mg; non-recommended 30 mg (“underdosed”) vs. recommended 60 mg. Most (22,166/26,823; 82.6%) patients received recommended doses. Non-recommended dosing was more frequent near label-specified dose-reduction thresholds. Ischemic stroke (IS; HR 0.85, 95% CI 0.50–1.47; *p* = 0.6) and major bleeding (MB; HR 1.47, 95% CI 0.97–2.71; *p* = 0.07) did not differ between recommended 60 mg and “underdosed” groups, whereas all-cause (HR 1.61, 95% CI 1.23–2.08; *p* = 0.0003) and cardiovascular deaths (HR 1.61, 95% CI 1.11–2.38; *p* = 0.01) were higher in the “underdosed” group. Compared with recommended 30 mg, the “overdosed” group had lower IS (HR 0.51, 95% CI 0.28–0.98; *p* = 0.04) and all-cause death (HR 0.74, 95% CI 0.55–0.98; *p* = 0.03) without higher MB (HR 0.74, 95% CI 0.46–1.22; *p* = 0.2). In conclusion: non-recommended dosing was infrequent, but more common near dose-reduction thresholds. “Underdosing” was not associated with better clinical outcomes. The “overdosed” group had lower IS and all-cause death without higher MB.

## 1. Introduction

Direct oral anticoagulants (DOACs) have replaced vitamin K antagonists (VKAs) as the standard of care for stroke prevention in patients with atrial fibrillation (AF) in most countries [1]. Label recommendations prescribing standard or reduced DOAC doses are product- and country-specific and are based on individual patient factors, such as renal function, body weight, use of specific concomitant medications, and age [2,3,4,5]. 

Dosing criteria for the DOACs were established based on data derived from pivotal phase III prospective, randomized, controlled clinical trials. Despite all reassuring data from these trials, prescriptions of non-recommended doses have been reported in a considerable percentage of patients in daily practice. Indeed, 25–50% of DOAC prescriptions were off-label according to a recent meta-analysis of 75 studies [6]. Among those patients receiving non-recommended doses, underdosing was more common than overdosing (9–68% vs. 3–36%). However, the frequency of non-recommended dosing seems to vary quite considerably between publications [6,7,8,9,10,11]. The fear of major hemorrhagic complications may lead to underdosing, which may be associated with a potential loss of effectiveness of the drugs, i.e., a higher risk of stroke and systemic embolic events (SEE) [8,12]. Compared with recommended dosing, overdosing could theoretically come at the price of higher incidence of major bleeding. 

Edoxaban 60 mg once daily (OD) is approved for prevention of stroke and SEE in patients with AF, with dose reduction to 30 mg OD recommended for patients who meet the following criteria: creatinine clearance (CrCl) 15–50 mL/min, low body weight (≤60 kg), and/or concomitant use of P-gp inhibitors [5]. In the United States (US), CrCl 15 to 50 mL/min is the sole criterion for dose reduction for AF patients [13]. Data on edoxaban dosing and its association with clinical outcomes in routine clinical practice have been very limited so far. To close this gap, the Global Edoxaban Treatment in routiNe clinical prActice (ETNA)-AF non-interventional program was devised to prospectively collect data from routine clinical practice on the characteristics, treatment adherence, treatment patterns, and outcomes of AF patients treated with edoxaban across Europe and Asia [14], and, thus, appears ideally suited to assess the effectiveness and safety in patients with AF receiving recommended/non-recommended edoxaban once-daily doses.

## 2. Materials and Methods

**Figure 1:**
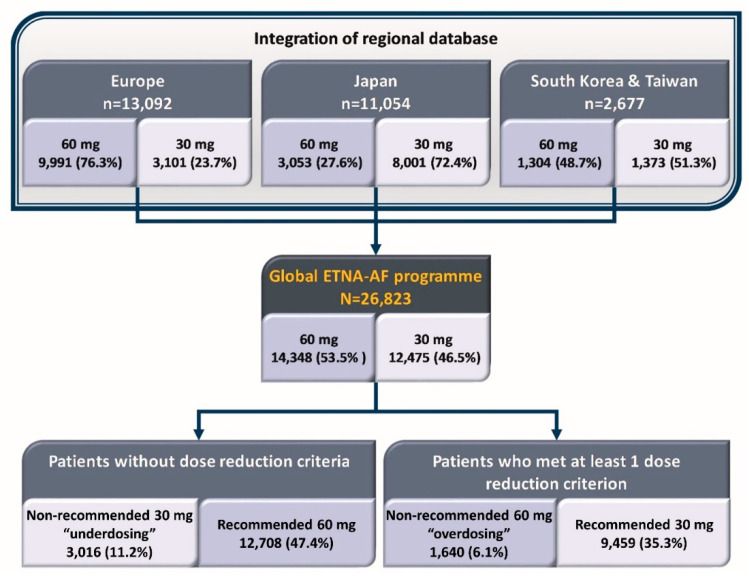
Patients included in the Global ETNA-AF non-interventional program.

The design of the Global ETNA-AF non-interventional program has been described previously [14]. The study, a prospective, observational, non-interventional study. was designed from the beginning to have data integrated from three jointly planned, separately operationalized studies conducted in Europe (Germany, Austria, Switzerland, Belgium, Italy, Spain, United Kingdom, Ireland, the Netherlands, and Portugal); Japan; and other Asian countries (Figure 1) [15]. The Global ETNA-AF protocols were approved prior to study initiation by the responsible ethics committees (EC) and institutional review boards (IRB). In Japan, the protocol was reviewed by the Pharmaceuticals and Medical Devices Agency prior to study initiation.

Eligible patients participating in Global ETNA-AF were those treated before enrolment with edoxaban for stroke prevention in AF according to the local labels. At the time of this study, there were no differences in dose reduction criteria between labels for the regions included in the analysis. In Japan, patients were only eligible for the study if they had received edoxaban for the first time to prevent ischemic stroke and SEE. Patients could not be included in the Global ETNA-AF program if they were participating simultaneously in any interventional study [14].

Patients’ medical and treatment history were collected at baseline. Clinical events captured at 12 months after enrolment included ischemic stroke, transient ischemic attack, myocardial infarction, SEE, bleeding, and all-cause and cardiovascular (CV)-related mortality; these events were reported based on physicians’ diagnoses and assessments per available guideline definitions. Events considered to be of special importance (e.g., major bleeding events, strokes, SEE, and deaths) were adjudicated by an independent clinical event adjudication committee [14]. No missing data were imputed, and only observed data were used.

In the present analysis, comparisons were made between:

- Those who received the non-recommended 60 mg (once daily; QD) dose vs. those receiving the recommended 30 mg (QD) dose, thus, assessing the effects in the “overdosed” group;
- And those who received the recommended edoxaban 60 mg (QD) dose vs. those receiving the non-recommended 30 mg (QD) dose, thus, assessing the effects in the “underdosed” group.

The term “recommended” dosing here used refers to the receipt of an edoxaban dose that is in accordance with the local edoxaban label (60 mg QD in patients who do not meet any of the aforementioned dose reduction criteria and 30 mg QD in patients who meet at least one of the criteria for reduction); conversely, the term “non-recommended” dosing refers to the receipt of an edoxaban dose that is not aligned with the local edoxaban label. The terms “underdosing/underdosed” and “overdosing/overdosed” are utilized herein for simplification and to align with terminology from other publications and, for this reason, are put in quotation marks; these terms do not imply that patients received an insufficient dose or a drug overexposure, respectively, which might translate to an unfavorable outcome. Patients receiving edoxaban 15 mg were excluded from this analysis.

### Statistical Analyses

Baseline data are presented descriptively as the mean with standard deviation (SD) for normal distributions, frequencies, and/or as summary statistics. The Wilcoxon two-sample test and chi-square test were applied for subgroup comparisons. Outcomes are reported with annual event rates and hazard ratios (HRs), both with 95% confidence intervals (CIs). HRs were calculated by using a Cox proportional hazard model and are reported along with their *p*-values. Stratification by dose in the Cox model and Kaplan–Meier curves is intended as a comparison of dosing subgroups and not as a measure of dose effect, as dosing decisions were at the clinician’s discretion.

The main goal of this study was to report the clinical outcomes related to “prescription patterns” of edoxaban dosing in daily practice rather than investigating the real impacts of dosing on risks of clinical events. Therefore, we did not aim to adjust for baseline characteristics between different dosing groups when comparing the risks of clinical events since the adjusted results would fail to demonstrate the real picture of clinical risks under different prescription patterns.

## 3. Results

### 3.1. Patient Characteristics

**Figure 2:**
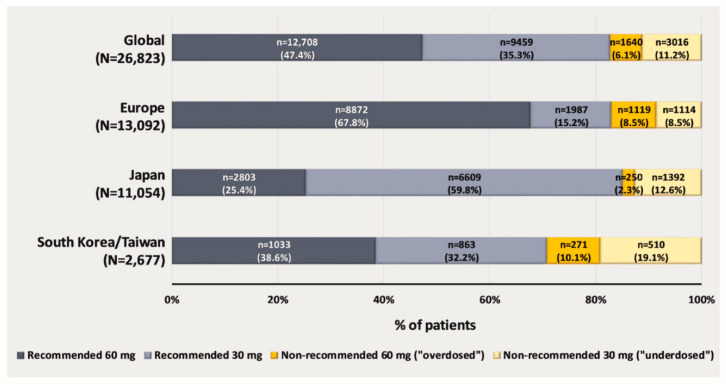
Edoxaban dosing * at baseline in the Global ETNA-AF non-interventional program. * Edoxaban dose reduction criteria: body weight ≤ 60 mg; creatinine clearance 15–50 mL/min; and concomitant use of certain P-gp inhibitors (ciclosporin, dronedarone, erythromycin, and ketoconazole).

Overall, 22, 166/26, and 823 (82.6%) of patients received a recommended dose (60 mg: 47.4%; 30 mg: 35.3%) (Figure 2). In Europe, most patients (76.3%) received edoxaban 60 mg, the majority of which was the recommended 60 mg dose (88.8%). In Japan, nearly three-quarters of patients (72.4%) received edoxaban 30 mg (recommended in 82.6%). In South Korea/Taiwan, similar proportions of patients received edoxaban 60 mg (48.7%) and 30 mg (51.3%), of whom the majority were dosed per label recommendations (79.2% and 62.9%, respectively) (Figure 2).

### 3.2. Patients without Dose Reduction Criteria 

The group of patients that received the non-recommended 30 mg dose (“underdosed”) vs. the group that received the recommended 60 mg dose was older (mean (SD) age: 74 (9.0) vs. 70 (9.3) years, *p* < 0.0001), had a lower CrCl (mean (SD) CrCl: 72.2 (20.7) vs. 85.8 (26.8) mL/min, *p* < 0.0001), and a greater proportion had long-standing persistent AF (15.0% vs. 6.8%) (Table 1). Patients who received the non-recommended 30 mg dose (“underdosed”) presented more often with cardiovascular (CV) comorbidities and risk factors such as diabetes (28.4% vs. 22.8%, *p* < 0.0001), myocardial infarction (4.8% vs. 3.4%, *p* = 0.0003), heart failure (20.6% vs. 13.2%, *p* < 0.0001) and ischemic stroke (11.0% vs. 8.6%, *p* < 0.0001), or history of major bleeding (2.1% vs. 1.1%, *p* < 0.0001) than those who received the recommended 60 mg dose (Table 1). Thus, the “underdosed” group presented with higher major bleeding risk and more often with CV comorbidities than the recommended 60 mg dose group. Significantly higher mean (SD) CHA_2_DS_2_-VASc (3.3 (1.5) vs. 2.8 (1.4), *p* < 0.0001) and HAS-BLED (2.5 (1.1) vs. 2.3 (1.1), *p* < 0.0001) scores were observed in the “underdosed” group compared to that of the recommended 60 mg group.

### 3.3. Patients with at Least 1 Dose Reduction Criterion

Patients who received the non-recommended 60 mg dose (“overdosed”) were younger (mean (SD) age: 75 [9.1] vs. 78 (8.5) years, *p* < 0.0001), had a higher CrCl (54.8 (20.5) vs. 49.6 (18.1) mL/min, *p* < 0.0001), and, in a smaller proportion, had long-standing persistent AF (6.7% vs. 16.7%) than the recommended 30 mg dose group. The mean (SD) CHA_2_DS_2_-VASc score at baseline was significantly lower in the “overdosed” group than the recommended 30 mg dose group (3.5 (1.4) vs. 3.9 (1.5), *p* < 0.0001; Table 1). The mean HAS-BLED score at baseline was similar in the two groups.

The “overdosed” group less frequently had a history of major bleeding (1.2% vs. 2.6%, *p* = 0.0007), ischemic stroke (10.2% vs. 16.4%, *p* < 0.0001), intracranial hemorrhage (0.8% vs. 2.1%, *p* = 0.0004) or comorbidities such as diabetes mellitus (20.4% vs. 22.7%, *p* = 0.04), heart failure (14.8% vs. 27.9%, *p* < 0.0001) and myocardial infarction (3.0% vs. 4.2%, *p* = 0.02) than the recommended 30 mg dose group (Table 1). Thus, the “overdosed” group was overall less sick than the recommended 30 mg dose group.

Patients receiving 60 mg were more likely to receive antiplatelet therapy than patients receiving 30 mg (recommended 60 mg vs. non-recommended 30 mg: 13.1% vs. 11.3%, *p* = 0.01; non-recommended 60 mg vs. recommended 30 mg: 12.7% vs. 8.0%, *p* < 0.0001) (Table 2). Heparin/fondaparinux was prescribed more often to recipients of recommended vs. non-recommended dosing (recommended 60 mg vs. non-recommended 30 mg: 9.0% vs. 5.8%, *p* < 0.0001; non-recommended 60 mg vs. recommended 30 mg: 8.8% vs. 11.3%, *p* = 0.01) (Table 2). The use of non-steroidal anti-inflammatory drugs (NSAIDs) and of P-gp inhibitors was low (<1.5%) for all dosing groups. 

**Figure 3:**
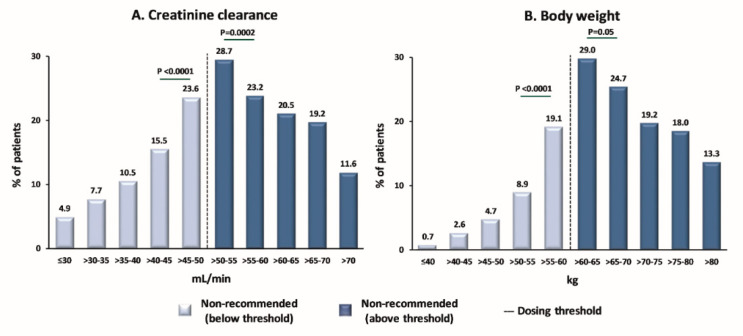
Proportion of patients receiving a non-recommended dose of edoxaban by (**A**) CrCl category or (**B**) body weight. The dashed vertical lines indicate the respective dose-reduction thresholds. The thresholds for dose reduction are CrCl ≤ 50 mL/min and body weight ≤ 60 kg, respectively. *p*-values are included for the category closest to the dosing threshold vs. the next closest category: CrCl 45–50 mL/min vs. CrCl 40–45 mL/min; CrCl 50–55 mL/min vs. CrCl 55–60 mL/min; body weight 55–60 kg vs. body weight 50–55 kg; body weight 60–65 kg vs. body weight 65–70 kg. CrCl, creatinine clearance.

In Europe, CrCl ≤ 50 mL/min was the most commonly met dose reduction criterion (59.8%) (Table S1), whereas, in Japan, a body weight of 60 kg or less was the key driver (74.2%) for administering edoxaban 30 mg (Table S1). In patients from South Korea/Taiwan, dose reduction to edoxaban 30 mg due to either low body weight (50.0%) or low CrCl (46.2%) was almost evenly split. Of all patients who received edoxaban 60 mg, 11.4% met the ≥1 dose reduction criterion and, therefore, should have been dose-reduced to 30 mg. Of the patients who received edoxaban 30 mg, 24.2% did not meet any dose reduction criteria and should have been treated with the full dose of edoxaban 60 mg. Importantly, non-recommended edoxaban use was significantly more frequent in patients near the dose reduction thresholds for CrCl (45–55 mL/min) or body weight (>55–65 kg) than in those further from the dose reduction thresholds (CrCl: >40–45 or >55–60 mL/min; body weight: >50–55 or >65–70 kg; Figure 3A,B).

### 3.4. Clinical Events 

**Figure 4:**
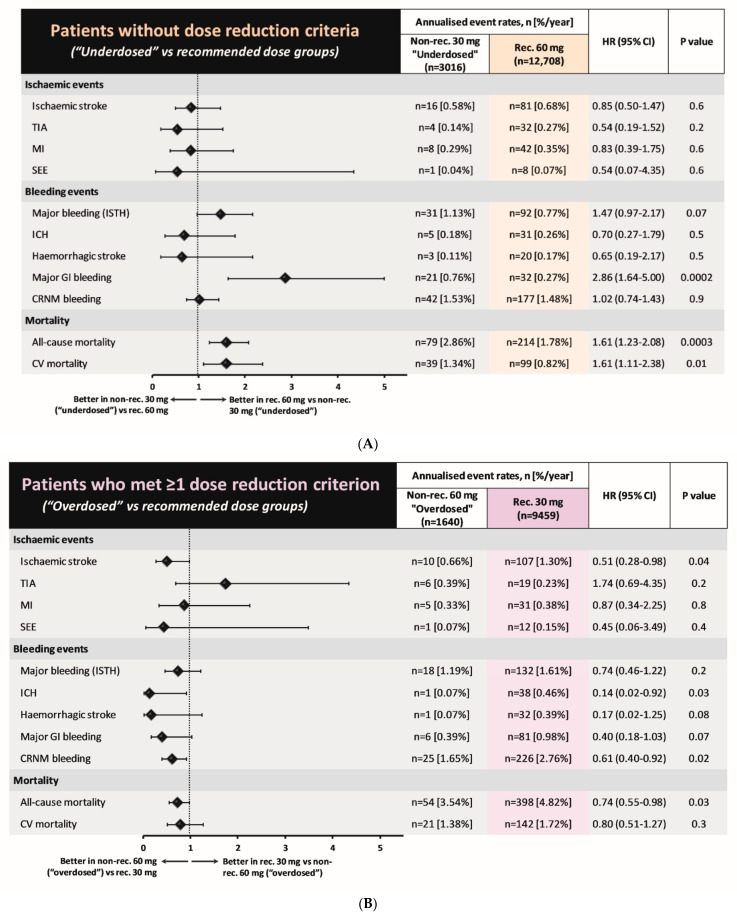
Forest plots of hazard ratios (95% CI) for clinical events in (**A**) patient groups without dose reduction criteria (“Underdosing” vs. recommended dosing) and (**B**) patient groups that met at least one dose reduction criterion (“Overdosing” vs. recommended dosing). CI, confidence interval; CRNM, clinically relevant non-major; CV, cardiovascular; HR, hazard ratio; ICH, intracranial hemorrhage; ISTH, International Society on Thrombosis and Hemostasis; MI, myocardial infarction; SEE, systemic embolic events; TIA, transient ischemic attack.

The rates of ischemic stroke (0.58%/year vs. 0.68%/year; HR 0.85, 95% CI 0.50–1.47; *p* = 0.6) and major bleeding (1.13%/year vs. 0.77%/year; HR 1.47, 95% CI 0.97–2.17; *p* = 0.07) did not differ significantly between “underdosed” and recommended 60 mg dosing groups, whereas rates of major gastrointestinal (GI) bleeding (0.76%/year vs. 0.27%/year; HR 2.86, 95% CI 1.64–5.00; *p* = 0.0002), all-cause death (2.86%/year vs. 1.78%/year; HR 1.61, 95% CI 1.23–2.08; *p* = 0.0003), and CV death (1.34%/year vs. 0.82%/year; HR 1.61, 95% CI 1.11–2.38; *p* = 0.01) were significantly higher in the “underdosed” group (Figure 4A).

In the “overdosed” group, the rate of ischemic stroke (0.66%/year vs. 1.30%/year; HR 0.51, 95% CI 0.28–0.98; *p* = 0.04) was lower and the rate of major bleeding (1.19%/year vs. 1.61%/year; HR 0.74, 95% CI 0.46–1.22; *p* = 0.2) was not significantly different from the recommended edoxaban 30 mg dose group (Figure 4B). The rate of all-cause death (3.54%/year vs. 4.82%/year; HR 0.74, 95% CI 0.55–0.98; *p* = 0.03) was significantly lower in the “overdosed” group compared with the recommended edoxaban 30 mg group, whereas there was no statistical difference in the rate of CV deaths (1.38%/year vs. 1.72%/year; HR 0.80, 95% CI 0.51–1.27; *p* = 0.3). The annualized ICH rates were 0.46%/year vs. 0.07%/year (HR 0.14, 95% CI 0.02–0.92; *p* = 0.03) in the recommended 30 mg dose group vs. the non-recommended 60 mg dose group (“overdosed”). If none of the criteria for edoxaban dose adjustment were fulfilled, then ICH annual rates did not differ (recommended 60 mg dose group vs. non-recommended 30 mg (“underdosed”) group, 0.26%/year vs. 0.18%/year; HR 0.70, 95% CI 0.27–1.79; *p* = 0.5).

**Figure 5:**
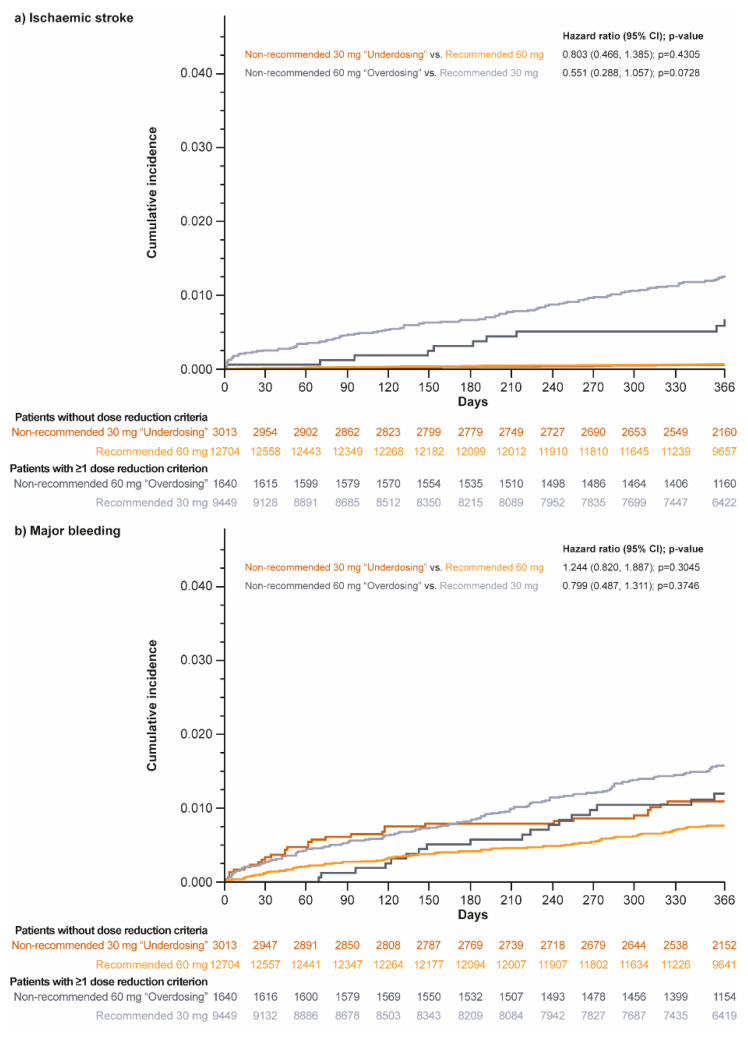

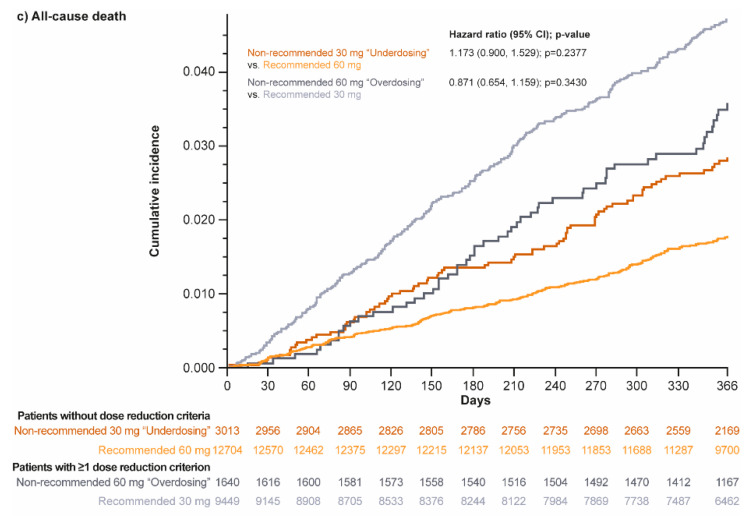
Kaplan–Meier curves for (**a**) ischemic stroke, (**b**) major bleeding, and (**c**) all-cause death in patients without dose reduction criteria (non-recommended 30 mg “underdosing” vs. recommended 60 mg dosing groups) and those who met at least one dose reduction criterion (non-recommended 60 mg “overdosing” vs. recommended 30 mg dosing groups).

Kaplan–Meier curves for ischemic stroke, major bleeding, and all-cause death are included in Figure 5.

## 4. Discussion

In the present study, we investigated the prescription patterns of edoxaban dosing and their associations with clinical outcomes in Global ETNA-AF, the largest real-world prospective registry dedicated to a single DOAC. Our principal findings are as follows: (i) non-recommended edoxaban use (either “under-” or “overdosing”) occurred in 17% of patients with AF, and was more frequent when patients were close to the threshold of dose reduction for CrCl (50 mL/min) or body weight (60 kg); (ii) compared with the recommended 60 mg dosing group, patients in the “underdosing” group were older, and with higher percentages of prior major bleeding or ischemic stroke; (iii) compared with the recommended 30 mg dosing group, patients in the “overdosing” group were younger, with lower percentages of prior major bleeding, including intracranial hemorrhage or ischemic stroke; (iv) compared with the recommended 60 mg dosing group, patients in the “underdosing” group had a higher rates of major GI bleeding and all-cause and CV death without higher ischemic stroke rates; and (v) compared with recommended 30 mg dosing group, patients in the “overdosing” group had lower rates of ischemic stroke without higher major bleeding rates. Non-recommended dosing was applied most often near the threshold for dose change, with apparent consequences in death outcomes in the “underdosed” group but no notable adverse consequences in either effectiveness or safety in the “overdosed” group.

### 4.1. Prescription Rates of Non-Recommended DOACs

In Global ETNA-AF, approximately 83% of patients were dosed according to label recommendations globally, with some regional differences (82.9%, 85.1%, and 70.8% in Europe, Japan, and South Korea/Taiwan, respectively). Of the overall population, 11.2% were “underdosed” and 6.1% were “overdosed”. Despite the small percentages of the overall 26,823 patients in this registry, both groups of “underdosed” and “overdosed” patients are relatively sizeable, consisting of 3016 and 1640 patients, respectively, allowing for meaningful interpretation. The recommended dosing rate in Global ETNA-AF (83%) was higher than that of previous reports (72.9% in GARFIELD-AF (Global Anticoagulant Registry in the FIELD-AF) [17], ~77% in APAF (APixaban in Atrial Fibrillation) registry [18], 80.8% in XANTUS [10], and 68% in Taiwan AF patients [11]). Edoxaban has been under-represented in observational studies published so far, as it was the last of the four DOACs to obtain regulatory approval. Our study provides important data regarding the dosing patterns of edoxaban in routine daily practice, which has never previously reported in such a robust fashion. 

### 4.2. Clinical Characteristics of Patients Receiving Non-Recommended Dosing DOACs

Clinical factors besides the dosage reduction criteria may have influenced physicians’ clinical decisions on edoxaban dosing. Compared with the group of patients who received the recommended dosing, patients in the “underdosed” group in Global ETNA-AF were older and presented with more CV comorbidities. They also were less frequently receiving antiplatelet agents and parenteral anticoagulants such as heparins or fondaparinux (Table 2), suggesting more caution by prescribing physicians in overloading them with antithrombotic agents, likely in a perception of higher frailty. Conversely, patients in the “overdosed” group were younger, relatively less sick, and less often had a history of major bleeding, and were more often receiving antiplatelet agents in concomitance with edoxaban (Table 2), suggesting lesser perceived frailty. These observations suggest that the “perceived” risk of bleeding for each individual as judged by physicians plays an important role in determining non-recommended dosing of edoxaban. Unsurprisingly, similar patterns were observed in other registries. In GARFIELD-AF, patients who received underdosed DOACs were older and had higher CHA_2_DS_2_-VASc scores compared with those who received a recommended dose [17]. Likewise, in a sub-analysis of XANTUS including 4464 patients on rivaroxaban, compared with patients on recommended doses both under- and overdosed patients had higher ischemic (CHADS_2_) and bleeding (HAS-BLED) scores, suggesting that patients perceived as high-risk for both stroke and bleeding were more likely to receive off-label DOAC doses [10].

Non-recommended edoxaban use was more frequent when patients were near the threshold of dose reduction for CrCl or body weight. Such finding suggests that, in patients close to the threshold for a dose change, physicians may replace the binary dose reduction criteria with other patient characteristics, attaining dosing decisions based on composite clinical evaluations of the overall patient characteristics.

### 4.3. Clinical Events Associated with Non-Recommended Dosing

An important medical concern is whether prescription of non-recommended DOAC dosing impacts clinical events. A recent sub-analysis of the ENGAGE AF-TIMI 48 trial showed a higher risk of stroke/SEE and lower risk of major bleeding, intracranial hemorrhage, major GI bleeding, and life-threatening bleeding in patients randomized to receive a reduced vs. standard dose of edoxaban [19]. In an analysis of the real-world GARFIELD-AF registry, patients receiving non-recommended DOAC doses (both under- and over-doses) were at a higher risk of all-cause death (HR 1.24, 95% CI 1.04–1.48; primarily CV death) than patients receiving recommended doses [17]. However, conclusions could not be drawn for edoxaban because of the small number of edoxaban-treated patients in that study (*n* = 286). Another analysis assessed DOAC dosing patterns and associated outcomes in a US claims database (*n* = 14,865) in patients who met the dose-reduction criteria (*n* = 1473) based on renal dysfunction: in this analysis, compared with standard doses of apixaban, those who were underdosed had a significantly higher risk of stroke (HR 4.87, 95% CI 1.30–18.26; *p* = 0.02) without the benefit of fewer major bleeding events [20]. Edoxaban data were not available in that report. A recent retrospective study from Taiwan reported the associations between off-label DOAC dosing and clinical outcomes among 11,275 patients, including 1483 patients treated with edoxaban [11]. The results showed that DOAC “underdosing” was associated with a higher risk of ischemic stroke/SEE in a pooled analysis of the four DOACs. For edoxaban, though, a higher risk of ischemic events was not detected for off-label dosing compared with the recommended dose (HR 1.43, 95% CI 0.53–3.89; *p* = 0.484). However, that study was limited by its retrospective design and the relatively low number of edoxaban patients. Data now reported in our Global ETNA-AF program, with a significantly greater number of patients (26,823 vs. 1483 in the Taiwanese study [11]), a prospective study design, and the adjudication of effectiveness and safety outcomes for approximately 50% of patients provide a much more robust basis for careful description of correlates and an initial attempt at interpretation.

Compared with patients who received the recommended 60 mg dose, the “underdosed” group did not have a higher rate of stroke/SEE events but did have a higher rate of major GI bleeding and all-cause and CV deaths. These clinical events might have been affected by factors other than stroke, such as older age, major bleeding history, and a higher prevalence of CV comorbidities. It is unlikely that concomitant medications such as NSAIDs, antiplatelets, and heparin contributed to the higher rate of major GI bleeding events in the “underdosed” group, as the baseline NSAIDs used in this subgroup were infrequent (0.3% of patients), and the baseline use of antiplatelets and heparin was actually significantly lower than use by the recommended 60 mg dose group. Patients in the “overdosed” group had a major bleeding event rate similar to that of patients who received the recommended edoxaban 30 mg dose, with lower rates of ischemic stroke and all-cause death in the “overdosed” group. In aggregate, these data suggest that, in real-world practice, clinicians’ dosing judgement/decisions and the subsequent clinical outcomes are often driven by patient characteristics that are not reflected in the label, especially for patients who are near the dose-reduction threshold. Further analyses of ETNA-AF with a longer (4-year) follow-up period will be performed to investigate the real impacts of edoxaban dosing on clinical outcomes after statistical adjustments for the differences in clinical characteristics between dosing groups out of the aims of the present report.

### 4.4. Strengths and Limitations

One critical strength of the Global ETNA-AF analysis is the large sample size of 26,823 patients: the present analysis is indeed the largest prospective source of routine-care data for a single DOAC in patients with AF. Global ETNA-AF also included a very diverse patient population, with more than 13,000 patients from Asia. In addition, data quality was enhanced by adjudicating efficacy and safety outcomes and calculating CrCl, a dose reduction criterion, with very few missing data points (8.5%). In contrast, nearly 35% of patients had missing CrCl values in the XANTUS registry [21], and CrCl was not routinely reported in GARFIELD-AF [17]. The real-world nature of this study offered the unique opportunity of examining the outcomes related to prescriptions of “underdosing” and “overdosing” edoxaban, as opposed to the strict dosing regimens allowed in randomized controlled trials. That recommended dosing leads to better results in these registry-based observations (with the potential implication that such recommendations should be therefore followed by the treating physician) is not an obvious finding. The choice of recommending 60 mg instead of 30 mg as the default dosing for edoxaban (in the absence of conditions demanding a dose reduction) has been itself a matter of debate, because the lower dosing regimen was associated with lower mortality compared with warfarin in the pivotal ENGAGE AF-TIMI 48, which was not the case for the higher dosing regimen. Therefore, a validation of this choice in a registry setting is of relevance and is indeed a strength of the overall ETNA-AF registry, requiring, however, further adjusted analyses, out of the scope of the present report. 

Notable limitations of this study include its one-armed observational design. Another limitation is that patients were grouped by edoxaban dose at baseline. As per the nature of the registry, it was not possible to control for changes in dosing and for adherence to the prescribed dose. In addition, we had no information on the duration of exposure to drugs throughout the study, which would have been useful to better assess the comparability of study groups. Ongoing analyses will allow adjustments for physicians’ characteristics, such as medical specialty and the site of prescription (e.g., hospital or outpatient clinic), among others, with a propensity score factoring all characteristics associated with inappropriate dosing. A small difference in “overdosed”/”underdosed” patient proportions is here reported within the three macro-regions where the study was run. Therefore, it is plausible that different reasons (e.g., creatinine clearance, body weight, or both) may have driven different dosage choices.

Finally, the eligibility criteria for the Global ETNA-AF program should be taken into consideration before extrapolating the present findings to DOAC- or edoxaban-naïve patients, as only patients with edoxaban experience were included to avoid any potential influence on physicians’ or patients’ decision making.

## 5. Conclusions

The present Global ETNA-AF study analysis suggests that physicians’ decisions for non-recommended dosing of edoxaban—while less frequent—are common near the dose reduction thresholds, with “underdosing” occurring more often than “overdosing”. When compared with patients who received the recommended edoxaban dosing, patients who were “underdosed” did not have better clinical outcomes, whereas “overdosed” patients did not have higher major bleeding rates and had lower rates of ischemic stroke and all-cause death.

## Figures and Tables

**Table 1:** Patient demographics, baseline characteristics, and medical history by dose reduction criteria cohort and edoxaban dose groups.

	Patients without Dose Reduction Criteria			Patients Who Met at Least 1 Dose Reduction Criterion		
	Recommended 60 mg (*n* = 12,708)	Non-Recommended 30 mg “Underdosed” (*n* = 3016)	*p* Value	Non-Recommended 60 mg “Overdosed” (*n* = 1640)	Recommended 30 mg (*n* = 9459)	*p*-Value
Age (y), mean (SD)	70 (9.3)	74 (9.0)	<0.0001	75 (9.1)	78 (8.5)	<0.0001
Age (y), *n* (%)						
<65	3024 (23.8)	395 (13.1)	<0.0001	184 (11.2)	530 (5.6)	<0.0001
65 to <75	5388 (42.4)	970 (32.2)		473 (28.8)	2338 (24.7)	
75 to <85	3919 (30.8)	1368 (45.4)		759 (46.3)	4365 (46.1)	
≥85	376 (3.0)	282 (9.4)		224 (13.7)	2226 (23.5)	
Male, *n* (%)	8962 (70.5)	2080 (69.0)	0.09	653 (39.8)	3910 (41.3)	0.2
Weight, kg, mean (SD)	81.8 (15.5)	76.0 (13.4)	<0.0001	64.1 (11.8)	56.1 (11.4)	<0.0001
BMI, kg/m^2^, mean (SD)	28.3 (4.8)	27.6 (4.4)	<0.0001	24.2 (3.9)	22.8 (3.7)	<0.0001
CrCl, * mL/min, mean (SD)	85.8 (26.8)	72.2 (20.7)	<0.0001	54.8 (20.5)	49.6 (18.1)	<0.0001
Type of AF, % (*n*)						
Paroxysmal	6573 (53.0)	1395 (48.3)	<0.0001	872 (54.3)	4465 (50.4)	<0.0001
Persistent	2860 (23.1)	497 (17.2)		329 (20.5)	1157 (13.1)	
Long-standing persistent	845 (6.8)	432 (15.0)		108 (6.7)	1476 (16.7)	
Permanent	1741 (14.0)	313 (10.8)		272 (16.9)	716 (8.1)	
CHA_2_DS_2_-VASc score, mean (SD) [median]	2.8 (1.4) [3.0]	3.3 (1.5) [3.0]	<0.0001	3.5 (1.4) [3.0]	3.9 (1.5) [4.0]	<0.0001
Modified HAS-BLED score, ^†^ mean (SD) [median]	2.3 (1.1) [2.0]	2.5 (1.1) [2.0]	<0.0001	2.5 (1.1) [2.0]	2.5 (1.1) [2.0]	0.4
Medical history, *n* (%)						
Hypertension	9519 (74.9)	2319 (76.9)	0.02	1192 (72.7)	6922 (73.2)	0.7
Diabetes mellitus	2903 (22.8)	858 (28.4)	<0.0001	335 (20.4)	2145 (22.7)	0.04
Coronary heart disease	1782 (18.0)	436 (26.8)	0.5	290 (20.9)	642 (22.5)	<0.0001
Myocardial infarction	430 (3.4)	144 (4.8)	0.0003	49 (3.0)	396 (4.2)	0.02
Heart failure ^‡^	1671 (13.2)	620 (20.6)	<0.0001	243 (14.8)	2638 (27.9)	<0.0001
Peripheral artery disease	262 (2.1)	89 (3.0)	0.003	55 (3.4)	226 (2.4)	0.02
COPD	773 (6.1)	174 (5.8)	0.5	124 (7.6)	344 (3.6)	<0.0001
Ischemic stroke	1093 (8.6)	333 (11.0)	<0.0001	167 (10.2)	1554 (16.4)	<0.0001
TIA	390 (3.1)	95 (3.1)	0.8	55 (3.4)	305 (3.2)	0.8
Major bleeding (ISTH)	134 (1.1)	63 (2.1)	<0.0001	20 (1.2)	247 (2.6)	0.0007
Intracranial hemorrhage	103 (0.8)	45 (1.5)	0.0005	13 (0.8)	196 (2.1)	0.0004
Major gastrointestinal bleeding	14 (0.1)	11 (0.4)	0.002	4 (0.2)	35 (0.4)	0.4

* Calculated using the Cockcroft–Gault formula. CrCl could not be calculated for 8.5% of patients due to missing data. ^†^ The modified HAS-BLED score was calculated without Labile INR. ^‡^ A patient was considered as having a medical history of heart failure if one of the following criteria was fulfilled: documented congestive heart failure; documented ischemic cardiomyopathy; documented ejection fraction < 40% [16]; frequent dyspnea (≥1/day) without chronic obstructive pulmonary disease, and at least one of the following: documented severe valvular heart disease, coronary heart disease post-myocardial infarction, valve replacement, or hypertension treated with ≥3 drugs [15]. AF, atrial fibrillation; BMI, body mass index; CHA_2_DS_2_-VASc, congestive heart failure, hypertension, age ≥ 75 (doubled), diabetes, stroke (doubled), vascular disease, age 65 to 74 and sex category; COPD, chronic obstructive pulmonary disease; CrCl, creatinine clearance; HAS-BLED, Hypertension, Abnormal renal/liver function, Stroke, Bleeding history or predisposition, Labile international normalized ratio, Elderly, Drugs/alcohol concomitantly; INR, international normalized ratio; IQR, interquartile range; ISTH, International Society on Thrombosis and Hemostasis; SD, standard deviation; TIA, transient ischemic attack; y, years.

**Table 2:** Concomitant medications at baseline.

Medication at Baseline, *n* (%)	Patients without Dose Reduction Criteria			Patients Who Met at Least 1 Dose Reduction Criterion		
	Recommended 60 mg (*n* = 12,708)	Non-Recommended 30 mg “Underdosing” (*n* = 3016)	*p* Value	Non-Recommended 60 mg “Overdosing” (*n* = 1640)	Recommended 30 mg (*n* = 9459)	*p*-Value
Antiplatelets	1660 (13.1)	342 (11.3)	0.01	208 (12.7)	759 (8.0)	<0.0001
Antiarrhythmic and rate control drugs	602 (5.4)	100 (4.6)	0.1	97 (6.5)	320 (5.8)	0.4
Heparin/fondaparinux	905 (9.0)	96 (5.8)	<0.0001	125 (8.8)	349 (11.3)	0.01
NSAIDs	13 (0.1)	5 (0.3)	0.1	1 (0.1)	23 (0.7)	0.004
P-gp inhibitors/inducers for which edoxaban dose adjustment is mandatory	10 (0.1)	3 (0.2)	0.4	3 (0.2)	44 (1.3)	0.0006
P-gp inhibitors/inducers for which edoxaban dose adjustment is not mandatory	4 (0)	3 (0.2)	0.04	3 (0.2)	13 (0.4)	0.4
Proton pump inhibitors	19 (0.2)	1 (0.1)	0.2	3 (0.2)	34 (0.8)	0.01
Hormone therapy	1 (0)	1 (0.1)	0.1	0 (0)	0 (0)	1.0

NSAIDs, non-steroidal anti-inflammatory drugs; P-gp, P-glycoprotein.

## Data Availability

The data underlying this article cannot be shared publicly as the Global ETNA-AF program is currently ongoing.

## Supplementary Materials

The following supporting information can be downloaded at: https://www.mdpi.com/article/10.3390/jcm12051870/s1, Table S1: Percentage of patients receiving edoxaban 60 mg and 30 mg doses according to local labels.
