# Supplementary material for: Prescribing Patterns and Outcomes of Edoxaban in Atrial Fibrillation: One-Year Data from the Global ETNA-AF Program
Source: J Clin Med. 2023 Feb 27;12(5):1870. doi: 10.3390/jcm12051870 (PMC10003604; doi:10.3390/jcm12051870)
Supplement: Supplementary file 1 [file jcm-12-01870-s001.zip › jcm-2214917-supplementary.pdf]

SUPPLEMENTARY DATA

Table S1. Percentage of patients receiving edoxaban 60 and 30 mg doses according to local labels

	Edoxaban Total (N=26,823)		Edoxaban 60 mg (n=14,348)		Edoxaban 30 mg (n=12,475)	
Body weight ≤60 kg	**n**	**%**	**n**	**%**	**n**	**%**
Europe	1310	10.3	488	5.1	822	27.2
South Korea/Taiwan	846	34.0	202	16.9	644	50.0
Japan	6000	55.2	168	5.6	5832	74.2
Global	8156	31.3	858	6.2	7298	60.0
Creatinine clearance 15–50 mL/min						
Europe	2391	21.0	708	8.3	1683	59.8
South Korea/Taiwan	704	29.7	133	11.8	571	46.2
Japan	3439	31.9	69	2.3	3370	43.3
Global	6534	26.7	910	7.2	5624	47.5
